# Supplementary material for: Enhancing market trend prediction using convolutional neural networks on Japanese candlestick patterns
Source: PeerJ Comput Sci. 2025 Feb 27;11:e2719. doi: 10.7717/peerj-cs.2719 (PMC11935771; doi:10.7717/peerj-cs.2719)
Supplement: Supplemental Information 1 [file peerj-cs-11-2719-s001.docx]

**Table 1.** Bullish Candlestick Patterns: Structural examples and components of candle types

| **Abandoned Baby** | **Belt-hold** | **Breakaway** | **Closing Marubozu** |
| --- | --- | --- | --- |
| 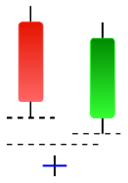 | 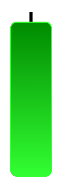 | 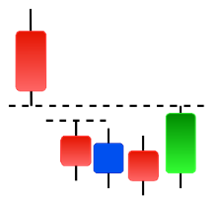 | 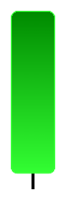 |
| **Concealing Baby Swallow** | **Counterattack** | **Doji Star** | **Dragonfly Doji** |
| 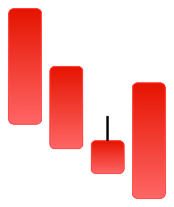 | 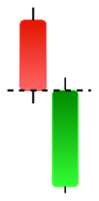 | 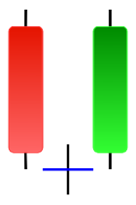 | 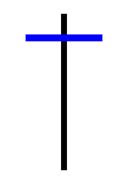 |
| **Engulfing** | **Hammer** | **Harami** | **Harami Cross** |
| 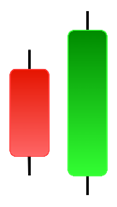 | 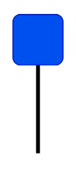 | 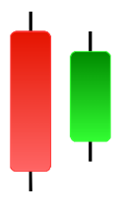 | 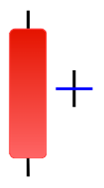 |
| **Homing Pigeon** | **Inverted Hammer** | **Kicking** | **Ladder Bottom** |
| 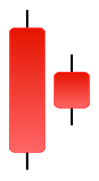 | 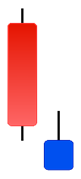 | 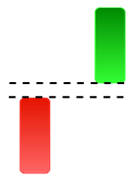 | 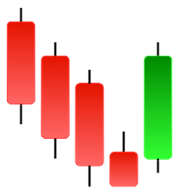 |
| **Long Line Candle** | **Marubozu** | **Mat Hold** | **Matching Low** |
| 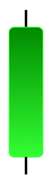 | 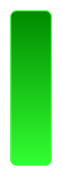 | 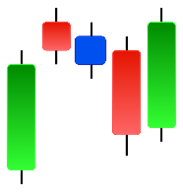 | 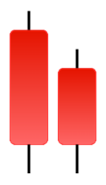 |
| **Morning Doji Star** | **Morning Star** | **Piercing Line** | **Rising Three Methods** |
| 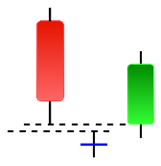 | 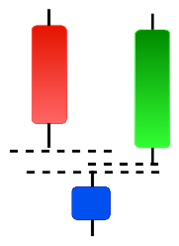 | 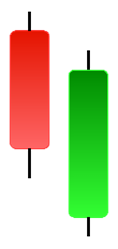 | 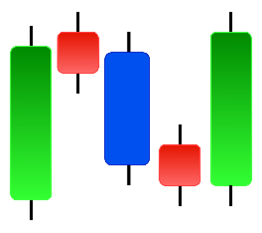 |
| **Separating Lines** | **Side by Side White Lines** | **Stick Sandwich** | **Takuri** |
| 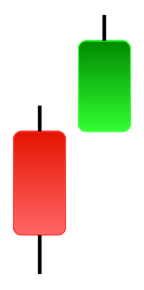 | 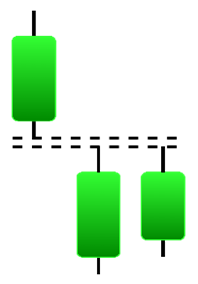 | 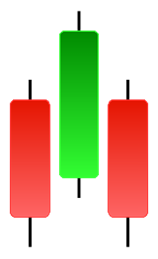 | 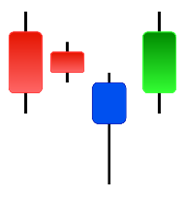 |
| **Tasuki Gap** | **Three Inside Up** | **Three Line Strike** | **Three Outside Up** |
| 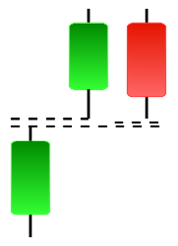 | 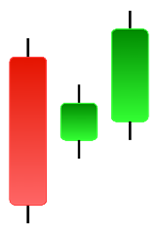 | 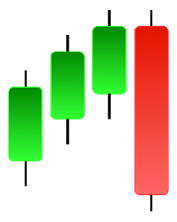 | 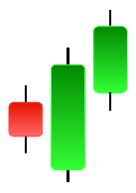 |
| **Three Stars in The South** | **Three White Soldiers** | **Tri-Star** | **Unique Three-River** |
| 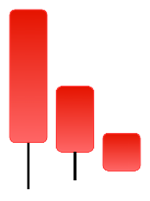 | 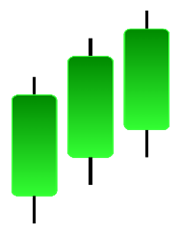 | 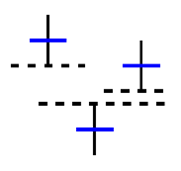 | 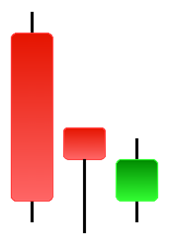 |
| **Upside Gap Three Methods** | | | |
| 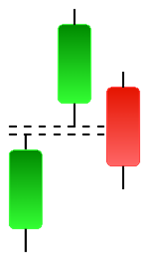 | | | |
