# Supplementary material for: Enhancing market trend prediction using convolutional neural networks on Japanese candlestick patterns
Source: PeerJ Comput Sci. 2025 Feb 27;11:e2719. doi: 10.7717/peerj-cs.2719 (PMC11935771; doi:10.7717/peerj-cs.2719)
Supplement: Supplemental Information 2 [file peerj-cs-11-2719-s002.docx]

**Table 2.** Bearish Candlestick Patterns: Structural examples and components of candle types

| **Abandoned Baby** | **Advance Block** | **Belt-hold** | **Breakaway** |
| --- | --- | --- | --- |
| 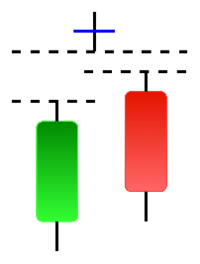 | 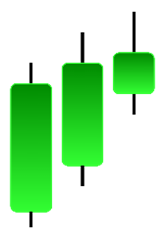 | 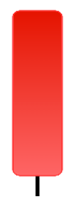 | 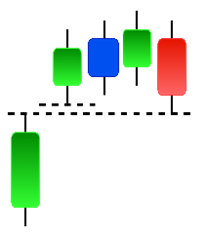 |
| **Closing Marubozu** | **Counterattack** | **Dark Cloud Cover** | **Deliberation** |
| 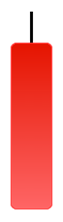 | 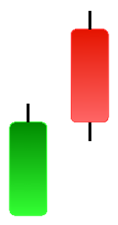 | 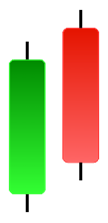 | 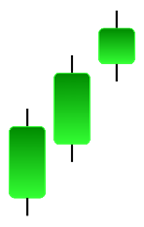 |
| **Doji Star** | **Downside Gap Three Methods** | **Engulfing** | **Evening Doji Star** |
| 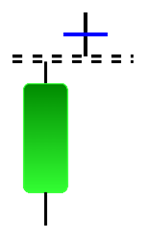 | 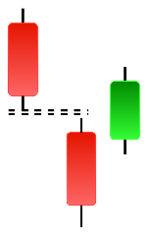 | 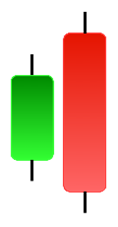 | 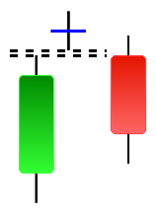 |
| **Evening Star** | **Falling Three Methods** | **Gravestone Doji** | **Hanging Man** |
| 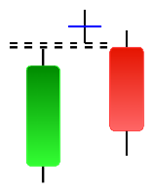 | 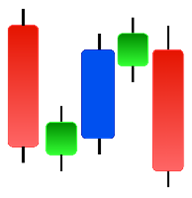 | 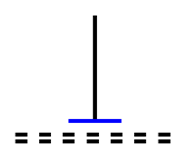 | 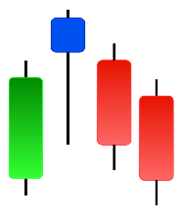 |
| **Harami** | **Harami Cross** | **In Neck** | **Kicking** |
| 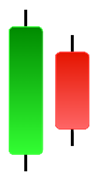 | 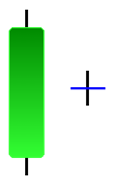 | 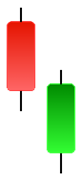 | 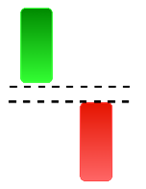 |
| **Long Line Candle** | **Marubozu** | **On Neck** | **Separating Lines** |
| 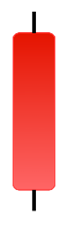 | 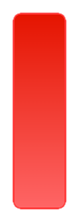 | 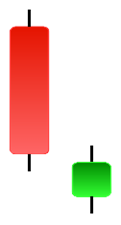 | 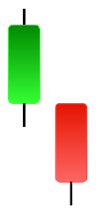 |
| **Shooting Star** | **Side by Side White Lines** | **Tasuki Gap** | **Three Black Crows** |
| 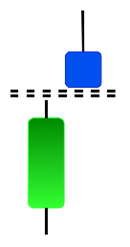 | 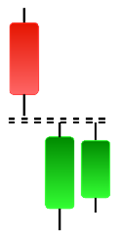 | 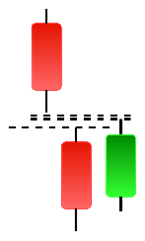 | 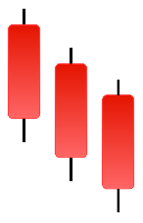 |
| **Three Inside Down** | **Three Line Strike** | **Three Outside Down** | **Thrusting** |
| 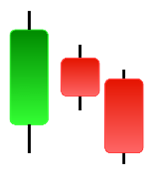 | 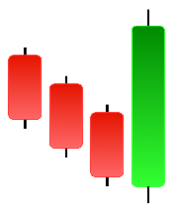 | 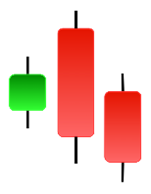 | 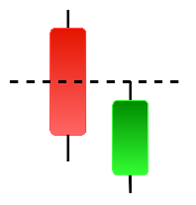 |
| **Tri-Star** | | **Upside Gap Two Crows** | |
| 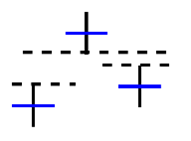 | | 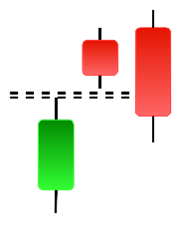 | |
