# Supplementary material for: Enhancing market trend prediction using convolutional neural networks on Japanese candlestick patterns
Source: PeerJ Comput Sci. 2025 Feb 27;11:e2719. doi: 10.7717/peerj-cs.2719 (PMC11935771; doi:10.7717/peerj-cs.2719)
Supplement: Supplemental Information 3 [file peerj-cs-11-2719-s003.docx]

**Table 3.** Architectural Details of the CNN Model

| **Layer Type and Configuration** | **Output Shape** | **Number of Parameters** | **Description and Functionality** |
| --- | --- | --- | --- |
| Input Layer | (None, 150, 150, 3) | 0 | Accepts input images with dimensions 150x150 pixels and 3 color channels (RGB). |
| Convolutional Layer 1 | (None, 148, 148, 32) | 896 | 32 filters of size (3x3), Activation: ReLU. Extracts basic edge features. |
| MaxPooling Layer 1 | (None, 74, 74, 32) | 0 | Pool Size: (2x2). Reduces spatial dimensions by half. |
| Convolutional Layer 2 | (None, 72, 72, 64) | 18,496 | 64 filters of size (3x3), Activation: ReLU. Captures higher-level features. |
| MaxPooling Layer 2 | (None, 36, 36, 64) | 0 | Pool Size: (2x2). Further downsampling for computational efficiency. |
| Convolutional Layer 3 | (None, 34, 34, 128) | 73,856 | 128 filters of size (3x3), Activation: ReLU. Detects complex patterns. |
| MaxPooling Layer 3 | (None, 17, 17, 128) | 0 | Pool Size: (2x2). Compresses feature maps while retaining important information. |
| Flatten Layer | (None, 36992) | 0 | Converts 3D feature maps into a 1D vector for fully connected layers. |
| Fully Connected Layer 1 | (None, 512) | 18,940,416 | Dense Layer: 512 units, Activation: ReLU. Learns high-level abstract features. |
| Dropout Layer | (None, 512) | 0 | Dropout Rate: 0.5. Mitigates overfitting by randomly deactivating nodes. |
| Output Layer | (N”one, 1) | 513 | Dense Layer: 1 unit, Activation: Sigmoid. Produces binary classification output. |
| **Total Parameters:** | | 19,034,177 (72.61 MB) | |
| **Trainable Parameters:** | | 19,034,177 (72.61 MB) | |
| **Non-trainable Parameters:** | | 0 (0.00 Byte) | |
