# Supplementary material for: Enhancing market trend prediction using convolutional neural networks on Japanese candlestick patterns
Source: PeerJ Comput Sci. 2025 Feb 27;11:e2719. doi: 10.7717/peerj-cs.2719 (PMC11935771; doi:10.7717/peerj-cs.2719)
Supplement: Supplemental Information 4 [file peerj-cs-11-2719-s004.docx]

**Table 4.** Training Hyperparameters and Compilation Parameters Used in the CNN Model.

| **Parameter** | **Value** |
| --- | --- |
| Input Image Dimensions | 150x150 |
| Batch Size | 64 |
| Image Rescaling Factor | 1/255 |
| Data Augmentation | Shear = 0.2, Zoom = 0.2 |
| Optimizer | Adam |
| Loss Function | Binary Crossentropy |
| Performance Metric | Accuracy |
| Number of Epochs | 20 |
| Learning Rate | 0.0003 |
