# Supplementary material for: Enhancing market trend prediction using convolutional neural networks on Japanese candlestick patterns
Source: PeerJ Comput Sci. 2025 Feb 27;11:e2719. doi: 10.7717/peerj-cs.2719 (PMC11935771; doi:10.7717/peerj-cs.2719)
Supplement: Supplemental Information 5 [file peerj-cs-11-2719-s005.docx]

**Table 5.** Frequency of various candlestick patterns within a 15-minute timeframe.

| **Pattern** | **Occurrences** | **Pattern** | **Occurrences** |
| --- | --- | --- | --- |
| Spinning Top | 19678 | Stalled Pattern | 346 |
| Long Line Candle | 19058 | Evening Star | 340 |
| Belt-hold | 17318 | Three-Line Strike | 337 |
| Short Line Candle | 15741 | Identical Three Crows | 250 |
| Closing Marubozu | 14445 | Morning Doji Star | 106 |
| Doji | 14133 | Evening Doji Star | 104 |
| Hikkake Pattern | 11894 | Modified Hikkake Pattern | 119 |
| High-Wave Candle | 11640 | Thrusting Pattern | 117 |
| Rickshaw Man | 9553 | Three Advancing White Soldiers | 86 |
| Engulfing Pattern | 7990 | Piercing Pattern | 76 |
| Marubozu | 5659 | Dark Cloud Cover | 68 |
| Harami Pattern | 4370 | Homing Pigeon | 47 |
| Three Outside Up/Down | 3854 | On-Neck Pattern | 32 |
| Hammer | 2776 | Stick Sandwich | 32 |
| Gravestone Doji | 2047 | In-Neck Pattern | 27 |
| Dragonfly Doji | 1942 | Tristar Pattern | 24 |
| Takuri (Dragonfly Doji with long lower shadow) | 1906 | Three Black Crows | 24 |
| Hanging Man | 1537 | Tasuki Gap | 19 |
| Matching Low | 1474 | Unique 3 River | 9 |
| Harami Cross Pattern | 1082 | Ladder Bottom | 7 |
| Doji Star | 967 | Breakaway | 4 |
| Three Inside Up/Down | 793 | Abandoned Baby | 1 |
| Upside/Downside Gap Three Methods | 719 | Two Crows | 1 |
| Shooting Star | 669 | Counterattack | 1 |
| Separating Lines | 624 |  |  |
