# Supplementary material for: Enhancing market trend prediction using convolutional neural networks on Japanese candlestick patterns
Source: PeerJ Comput Sci. 2025 Feb 27;11:e2719. doi: 10.7717/peerj-cs.2719 (PMC11935771; doi:10.7717/peerj-cs.2719)
Supplement: Supplemental Information 6 [file peerj-cs-11-2719-s006.docx]

**Table 6.** The performance results according to common metrics obtained from the implemented model.

| **Window Size** | **Shift Size** | **Precision** | **Recall** | **F1-Score** | **Accuracy** |
| --- | --- | --- | --- | --- | --- |
| **5** | **2** | **0.993** | **0.993** | **0.993** | **0.993** |
| 10 | 5 | 0.977 | 0.977 | 0.977 | 0.977 |
| 15 | 7 | 0.988 | 0.988 | 0.988 | 0.988 |
| 20 | 10 | 0.969 | 0.969 | 0.969 | 0.969 |
| 25 | 12 | 0.982 | 0.982 | 0.982 | 0.982 |
| 30 | 15 | 0.974 | 0.974 | 0.974 | 0.974 |
