# Supplementary material for: Enhancing market trend prediction using convolutional neural networks on Japanese candlestick patterns
Source: PeerJ Comput Sci. 2025 Feb 27;11:e2719. doi: 10.7717/peerj-cs.2719 (PMC11935771; doi:10.7717/peerj-cs.2719)
Supplement: Supplemental Information 7 [file peerj-cs-11-2719-s007.docx]

**Table 7.** Performance Evaluation of Loss and Accuracy Across Multiple Configurations and Cross-Validation Folds

Fold Config 1: W=5, S=2 Config 2: W=10, S=5 Config 3: W=15, S=7 Config 4: W=20, S=10 Config 5: W=25, S=12 Config 6: W=30, S=15

|  | Loss | Acc |  | Loss | Acc |  | Loss | Acc |  | Loss | Acc |  | Loss | Acc |  | Loss | Acc |  |
| --- | --- | --- | --- | --- | --- | --- | --- | --- | --- | --- | --- | --- | --- | --- | --- | --- | --- | --- |
| 1 | 0.0293 | 0.9929 |  | 0.0284 | 0.9879 |  | 0.0170 | 0.9933 |  | 0.1002 | 0.9633 |  | 0.0481 | 0.9855 |  | 0.0385 | 0.9875 |  |
| 2 | 0.0124 | 0.9944 |  | 0.0604 | 0.9829 |  | 0.1131 | 0.9816 |  | 0.0770 | 0.9714 |  | 0.0536 | 0.9801 |  | 0.0658 | 0.9817 |  |
| 3 | 0.0374 | 0.9873 |  | 0.0461 | 0.9889 |  | 0.0428 | 0.9906 |  | 0.0240 | 0.9932 |  | 0.0753 | 0.9839 |  | 0.0845 | 0.9788 |  |
| 4 | 0.0339 | 0.9913 |  | 0.0601 | 0.9789 |  | 0.0260 | 0.9924 |  | 0.0642 | 0.9796 |  | 0.0466 | 0.9870 |  | 0.0990 | 0.9624 |  |
| 5 | 0.0140 | 0.9936 |  | 0.0659 | 0.9819 |  | 0.0256 | 0.9933 |  | 0.0503 | 0.9850 |  | 0.0643 | 0.9748 |  | 0.0636 | 0.9826 |  |
| **Average** | **0.0254** | **0.9919** |  | **0.0522** | **0.9841** |  | **0.0449** | **0.9902** |  | **0.0631** | **0.9785** |  | **0.0576** | **0.9823** |  | **0.0703** | **0.9786** |  |
