# Supplementary material for: Enhancing market trend prediction using convolutional neural networks on Japanese candlestick patterns
Source: PeerJ Comput Sci. 2025 Feb 27;11:e2719. doi: 10.7717/peerj-cs.2719 (PMC11935771; doi:10.7717/peerj-cs.2719)
Supplement: Supplemental Information 8 [file peerj-cs-11-2719-s008.docx]

**Table 8.** The summary of Average Accuracy and Loss for Each Configuration

**Configuration Window Size Shift Average Loss Average Accuracy**

| Config 1 | 5 | 2 | 0.0254 | 0.9919 |
| --- | --- | --- | --- | --- |
| Config 2 | 10 | 5 | 0.0522 | 0.9841 |
| Config 3 | 15 | 7 | 0.0449 | 0.9902 |
| Config 4 | 20 | 10 | 0.0631 | 0.9785 |
| Config 5 | 25 | 12 | 0.0576 | 0.9823 |
| Config 6 | 30 | 15 | 0.0703 | 0.9786 |
