# Supplementary material for: Enhancing market trend prediction using convolutional neural networks on Japanese candlestick patterns
Source: PeerJ Comput Sci. 2025 Feb 27;11:e2719. doi: 10.7717/peerj-cs.2719 (PMC11935771; doi:10.7717/peerj-cs.2719)
Supplement: Supplemental Information 9 [file peerj-cs-11-2719-s009.docx]

**Table 9.** Performance Metrics of Pre-trained CNN Models.

| **CNN Model** | **Precision** | **Recall** | **F1-Score** | **Accuracy** |
| --- | --- | --- | --- | --- |
| VGG19 | 0.927 | 0.926 | 0.925 | 0.925 |
| VGG16 | 0.925 | 0.925 | 0.925 | 0.925 |
| ResNet50 | 0.912 | 0.908 | 0.907 | 0.907 |
| MobileNet | 0.935 | 0.933 | 0.933 | 0.933 |
| EfficientNetB0 | 0.922 | 0.921 | 0.920 | 0.920 |
| InceptionResNetV2 | 0.255 | 0.500 | 0.337 | 0.509 |
| MobileNetV2 | 0.907 | 0.907 | 0.907 | 0.907 |
| DenseNet121 | 0.863 | 0.809 | 0.804 | 0.812 |
| InceptionV3 | 0.690 | 0.592 | 0.528 | 0.586 |
