# Supplementary material for: Enhancing market trend prediction using convolutional neural networks on Japanese candlestick patterns
Source: PeerJ Comput Sci. 2025 Feb 27;11:e2719. doi: 10.7717/peerj-cs.2719 (PMC11935771; doi:10.7717/peerj-cs.2719)
Supplement: Supplemental Information 10 [file peerj-cs-11-2719-s010.docx]

**Table 10.** The comprehensive comparison of the related studies using Candlestick Charts.

| **Authors** | **Stock Market** | **Time Frame** | **Model Used** | **Input Dataset** | **Classification** | **Accuracy** |
| --- | --- | --- | --- | --- | --- | --- |
| [Lin et al.](#_bookmark40) [(2021)](#_bookmark40) | China’s | Daily | RF, GBDT, LR, KNN, | Historical stock prices, | Categorical | 60% |
|  |  |  | SVM, LSTM | technical indicators |  |  |
| [Hung and Chen](#_bookmark36) | Taiwan and Tokyo | Daily | CNN-autoencoder, | Candlestick Charts | Binary | 84% |
| [(2021)](#_bookmark36) |  |  | RNN |  |  |  |
| [Trang-Thi Ho and](#_bookmark52) | Apple, Tesla, IBM, | Daily | 1-D CNN and 2-D | Candlestick charts + | Binary | 75.38% |
| [Yennun Huang](#_bookmark52) | Amazon, and Google |  | CNN | Twitter Text |  |  |
| [(2021)](#_bookmark52) |  |  |  |  |  |  |
| [Ardiyanti et al.](#_bookmark23) | IDX | Daily | ANN+K-Fold Cross- | Candlestick Pattern | Binary | 85.96% |
| [(2021)](#_bookmark23) |  |  | Validation | data |  |  |
| [Chen and Tsai](#_bookmark29) [(2022)](#_bookmark29) | Foreign exchange | 1-minute | YOLO model | GAF encoded candle- | Binary | 88.35% |
|  | (EUR/USD) |  |  | stick charts |  |  |
| [Liang et al.](#_bookmark39) [(2022)](#_bookmark39) | China’s | Daily | K-line | K-line patterns | Binary | 56.04% and |
|  |  |  |  |  |  | 55.56% |
| [Santur](#_bookmark50) [(2022)](#_bookmark50) | 11 world indices | Daily | Ensemble Learning- | Candlestick Chart | Binary | 53.8% |
|  |  |  | Xgboost |  |  |  |
| [Wang et al.](#_bookmark54) [(2022)](#_bookmark54) | China’s (CSI 300) | Daily | Graph Neural Network | Candlestick is repre- | Categorical | - |
|  |  |  |  | sented by graph em- |  |  |
|  |  |  |  | bedding |  |  |
| [Behar and Sharma](#_bookmark25) | Indian (BSE and | Daily | KNN | Candlestick charts | Binary | 61.4% |
| [(2022)](#_bookmark25) | NIFTY 50) and US |  |  |  |  |  |
|  | (S&P500 and DJIA) |  |  |  |  |  |
| [Ramadhan et al.](#_bookmark47) | Nasdaq100 | Hourly | CNN-LSTM | GAF encoded candle- | Binary | 90% and 93% |
| [(2022)](#_bookmark47) |  |  |  | stick charts |  |  |
| [Puteri et al.](#_bookmark46) [(2023)](#_bookmark46) | Forex (GBP/USD) | 4-hour | SVM | OHLC candlestick | Binary | 90.72% |
|  |  |  |  | data |  |  |
| [Ruixun Zhang and](#_bookmark49) | Exchange-traded funds | Daily | Channel and Spatial- | Candlestick charts | Binary | Sharpe ratios |
| [Lin](#_bookmark49) [(2023)](#_bookmark49) | (ETF) |  | Attention CNN (CS- |  |  | between 1.57 |
|  |  |  | ACNN) |  |  | and 3.03 |
| [Vijayababu et al.](#_bookmark53) | Ahihi Dataset | Daily | VGG16, ResNet50, | OHLC candlestick pat- | Binary | 91.51% |
| [(2023)](#_bookmark53) |  |  | AlexNet, GoogleNet, | tern |  |  |
|  |  |  | YOLOv8 |  |  |  |
| [Chen et al.](#_bookmark31) [(2024)](#_bookmark31) | Chinese | Daily | Bidirectional GRU | OHLC candlestick | Categorical | - |
|  |  |  | with Candlestick | data |  |  |
|  |  |  | Patterns and Sparrow |  |  |  |
|  |  |  | Search Algorithm |  |  |  |
|  |  |  | (SSA-CPBiGRU) |  |  |  |
| [Huang et al.](#_bookmark35) [(2024)](#_bookmark35) | Chinese (Kweichow | Daily | Vector auto-regression | OHLC candlestick | Binary | - |
|  | Moutai, CSI 100, and |  | (VAR), Vector error | data |  |  |
|  | 50 ETF) |  | correction model |  |  |  |
|  |  |  | (VECM) |  |  |  |
| **Proposed Model** | **Forex (EUR/USD)** | **15-minute** | **CNN** | **Candlestick charts** | **Binary** | **99.3%** |
