# Supplementary material for: Enhancing market trend prediction using convolutional neural networks on Japanese candlestick patterns
Source: PeerJ Comput Sci. 2025 Feb 27;11:e2719. doi: 10.7717/peerj-cs.2719 (PMC11935771; doi:10.7717/peerj-cs.2719)
Supplement: Supplemental Information 11 [file peerj-cs-11-2719-s011.docx]

**Table 11.** CNN Model Complexity Analysis

| **Model Overview** |  | | |
| --- | --- | --- | --- |
| Total Parameters: |  | 19,034,177 |  |
| Total FLOPs: |  | 221,008,385 |  |
| Parameter Memory: |  | 72.61 MB |  |
| Model Size: |  | 72.65 MB |  |
| Model Depth: |  | 5 Layers |  |
| **Layer-wise Analysis** |  |  |  |
| **Layer Type** | **Parameters** | **FLOPs** | **Output Shape** |
| Conv2D | 896 | 19,625,984 | (64, 148, 148, 32) |
| MaxPooling2D | 0 | 700,928 | (64, 72, 72, 32) |
| Conv2D | 18,496 | 95,883,264 | (64, 72, 72, 64) |
| MaxPooling2D | 0 | 331,776 | (64, 36, 36, 64) |
| Conv2D | 73,856 | 85,377,536 | (64, 34, 34, 128) |
| MaxPooling2D | 0 | 147,968 | (64, 17, 17, 128) |
| Flatten | 0 | 0 | (64, 36992) |
| Dense | 18,940,416 | 18,940,416 | (64, 512) |
| Dropout | 0 | 0 | (64, 512) |
| Dense | 513 | 513 | (64, 1) |

**Inference Time Analysis**

Mean Inference Time: 570.20 ms

Std Inference Time: 84.14 ms

Min Inference Time: 397.73 ms

Max Inference Time: 741.64 ms
